# Comparative Analysis of the Chloroplast Genomes of the Chinese Endemic Genus *Urophysa* and Their Contribution to Chloroplast Phylogeny and Adaptive Evolution

**DOI:** 10.3390/ijms19071847

**Published:** 2018-06-22

**Authors:** Deng-Feng Xie, Yan Yu, Yi-Qi Deng, Juan Li, Hai-Ying Liu, Song-Dong Zhou, Xing-Jin He

**Affiliations:** Key Laboratory of Bio-Resources and Eco-Environment of Ministry of Education, College of Life Sciences, Sichuan University, Chengdu 610065, Sichuan, China; df_xie2017@163.com (D.-F.X.); yyu@scu.edu.cn (Y.Y.); yiqiden@gmail.com (Y.-Q.D.); lijuanxxn@163.com (J.L.); lhy921180@163.com (H.-Y.L.); songdongzhou@aliyun.com (S.-D.Z.)

**Keywords:** *Urophysa*, *Semiaquilegia adoxoides*, cp genome, repeat analysis, SSRs, positive selection analysis, phylogeny

## Abstract

*Urophysa* is a Chinese endemic genus comprising two species, *Urophysa rockii* and *Urophysa henryi*. In this study, we sequenced the complete chloroplast (cp) genomes of these two species and of their relative *Semiquilegia adoxoides*. Illumina sequencing technology was used to compare sequences, elucidate the intra- and interspecies variations, and infer the phylogeny relationship with other Ranunculaceae family species. A typical quadripartite structure was detected, with a genome size from 158,473 to 158,512 bp, consisting of a pair of inverted repeats separated by a small single-copy region and a large single-copy region. We analyzed the nucleotide diversity and repeated sequences components and conducted a positive selection analysis by the codon-based substitution on single-copy coding sequence (CDS). Seven regions were found to possess relatively high nucleotide diversity, and numerous variable repeats and simple sequence repeats (SSR) markers were detected. Six single-copy genes (*atpA*, *rpl20*, *psaA*, *atpB*, *ndhI*, and *rbcL*) resulted to have high posterior probabilities of codon sites in the positive selection analysis, which means that the six genes may be under a great selection pressure. The visualization results of the six genes showed that the amino acid properties across each column of all species are variable in different genera. All these regions with high nucleotide diversity, abundant repeats, and under positive selection will provide potential plastid markers for further taxonomic, phylogenetic, and population genetics studies in *Urophysa* and its relatives. Phylogenetic analyses based on the 79 single-copy genes, the whole complete genome sequences, and all CDS sequences showed same topologies with high support, and *U. rockii* was closely clustered with *U. henryi* within the *Urophysa* genus, with *S. adoxoides* as their closest relative. Therefore, the complete cp genomes in *Urophysa* species provide interesting insights and valuable information that can be used to identify related species and reconstruct their phylogeny.

## 1. Introduction

The genus *Urophysa* (Ranunculaceae) is a Chinese endemic genus with only two species, *Urophysa rockii* Ulbr. and *Urophysa henryi* (Oliv.) Ulbr. *U. rockii* is an extremely rare species with fewer than 2000 individuals living in Jiangyou, a Sichuan province of China, and *U. henryi* is distributed in Guizhou, south Chongqing, north Hunan, and west Hubei [1]. The two species’ natural populations are restricted to small and isolated areas separated by high mountains and deep valleys and grow in steep and karstic cliffs with dramatically shrinking and fragmenting natural distributions [2]. In addition, the plants are collected for Chinese traditional medicine for the treatment of contusions and bruises, which contributed to the decline of their populations [3]. Previous studies on the genus *Urophysa* are scarce and mainly focused on the endangered *U. rockii*, its growing environment and conservation strategies [4], its biological and ecological characteristics, and its reproductive biology [5,6]. A recent study suggested that the uplift of the Yungui Plateau played an important role in the species divergence of *Urophysa* [2]. However, the chloroplast DNA (cpDNA) phylogeny showed inconsistency with the nuclear ribosomal DNA (nrDNA). Hence, to gain a better insight into the relationship of these two species and understand their genome structure so as to facilitate their speciation process and the conservation of *U. rockii*, we assembled and characterized the complete chloroplast genome sequence of *U. rockii* and *U. henryi* using the Illumina paired-end sequencing reads.

The angiosperm cp genome is one of the three DNA genomes (the other two are nuclear and mitochondrial genome), is uniparentally inherited, and has a high conserved circular DNA arrangement [7]. It is widely considered an informative and valuable resource for investigating evolutionary biology because of its relatively stable genome structure, gene content, and gene order [8,9,10,11,12,13]. The cp genome of plants always ranges from 115 to 210 kb and has a quadripartite structure that is typically composed of two copies of inverted repeat (IR) regions, which are separated by a large single-copy (LSC) region and a small single-copy (SSC) region [14,15,16]. Because of its compact size, less recombination, and maternal inheritance, the cp genome has been used to generate genetic markers for phylogenetic analysis [17,18], molecular identification [19], and divergence dating [20]. Especially, the low evolutionary rate of the cp genome in taxa that are not very young makes it an ideal system for assessing plant phylogeny [21].

In the present study, we report the complete chloroplast genome sequences of these two *Urophysa* species and their relative *Semiquilegia adoxoides* for the first time. Combining previously reported cp genome sequences, we performed phylogenetic analyses according to the whole cp genome and shared single-copy genes. Our findings will contribute to our understanding of the evolutionary history of the genus *Urophysa*. Additionally, highly variable regions and genes that were detected to be under positive selection could be employed to develop potential markers for phylogenetic analyses or candidates for DNA barcoding in future studies.

## 2. Results and Discussion

### 2.1. Complete Chloroplast Genomes of Three Species

**Figure 1:**
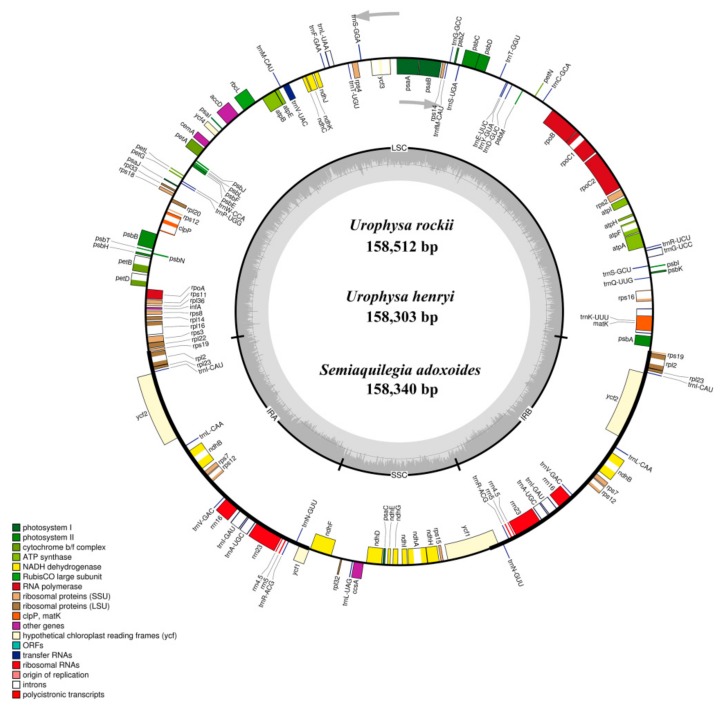
Gene maps of the *Urophysa rockii*, *Urophysa henryi* and *Semiquilegia adoxoides* chloroplast (cp) genomes. Genes shown inside the circle are transcribed clockwise, and those outside are transcribed counterclockwise. Genes belonging to different functional groups are color-coded. The darker gray color in the inner circle corresponds to the GC content, and the lighter gray color corresponds to the AT content. SSU: small subunit; LSU: large subunit; ORF: open reading frame.

The complete chloroplast genome of *U. rockii*, *U. henryi*, and *S. adoxoides* showed a single circular molecule with a typical quadripartite structure (Figure 1). The sizes of the *U. rockii*, *U. henry*, and *S. adoxoides* cp genomes were found to be 158,512 bp, 158,303, and 158,340 bp, respectively, which are in the range of most angiosperm plastid genomes [22]. The cp genome consists of a pair of IRs (IRa and IRb, with length 26,473–26,584 bp), separated by a LSC (87,031–87,202 bp) region and one SSC (18,192–18,220 bp) region (Table 1). The GC content of each species was very similar in the whole cp genome and the same region (LSC, SSC, and IR), but in the IR regions it was clearly higher than in the other regions, possibly because of the high GC content of the rRNA (55.8%) that was located in the IR regions (Table 2). These results are similar to a previously reported high GC percentage in IR regions [23,24,25].

The genomes contain 87 coding genes, 36 transfer RNA genes (tRNA), and 8 ribosomal RNA genes (rRNA) (Table 3). Most of the genes occur as a single copy in LSC or SSC regions, while 18 genes are duplicated in the IR regions, including seven protein-coding genes (*ndhB*, *rpl2*, *rpl23*, *rps7*, *rps12*, *rps19*, *ycf2*), seven tRNA species (*trnA-UGC*, *trnI-CAU*, *trnI-GAU*, *trnL-CAA*, *trnN-GUU*, *trnR-ACG*, and *trnV-GAC*) and four rRNA species (*rrn4.5*, *rrn5*, *rrn16*, and *rrn23*). The gene *ycf1* straddles the SSC and IRs, while *rps12* locates its first exon in the LSC region and two other exons in the IRs. The LSC region comprises 63 protein-coding genes and 21 tRNA genes, whereas the SSC and IR regions include 12 and 7 protein-coding genes, with one and seven tRNA, respectively. The protein-coding genes present in the *U. rockii* cp genome include 9 genes encoding large ribosomal proteins (*rpl2*, *rpl14*, *rpl16*, *rpl20*, *rpl22*, *rpl23*, *rpl32*, *rpl33*, *rpl36*) and 12 genes encoding small ribosomal proteins (*rps2*, *rps3*, *rps4*, *rps7*, *rps8*, *rps11*, *rps12*, *rps14*, *rps15*, *rps16*, *rps18*, *rps19*). There are 5 genes encoding phytosystem I subunits (*psaA*, *psaB*, *psaC*, *psaI*, *psaJ*), along with 15 genes related to photosystem II subunits (*psbA*, *psbB*, *psbC*, *psbD*, *psbE*, *psbF*, *psbH*, *psbI*, *psbJ*, *psbK*, *psbL*, *psbM*, *psbN*, *psbT*, *psbZ*) (Table 3). Six genes (*atpA*, *atpB*, *atpE*, *atpF*, *atpH*, *atpI*) encode ATP synthase and electron transport chain components (Table 3). A similar pattern of protein-coding genes is also present in *U. henryi* and *S. adoxoides*. There are eight intron-containing genes, six of which contain one intron; only the genes *clpP* and *ycf3* have two introns (Table S1). All these eight genes possess at least two exons, and *ycf3* has three exons. The *rps16* gene has the longest intron (866 bp), and *rpoC1* has the longest exon (1613 bp).

### 2.2. Repeat Analysis

**Figure 2:**
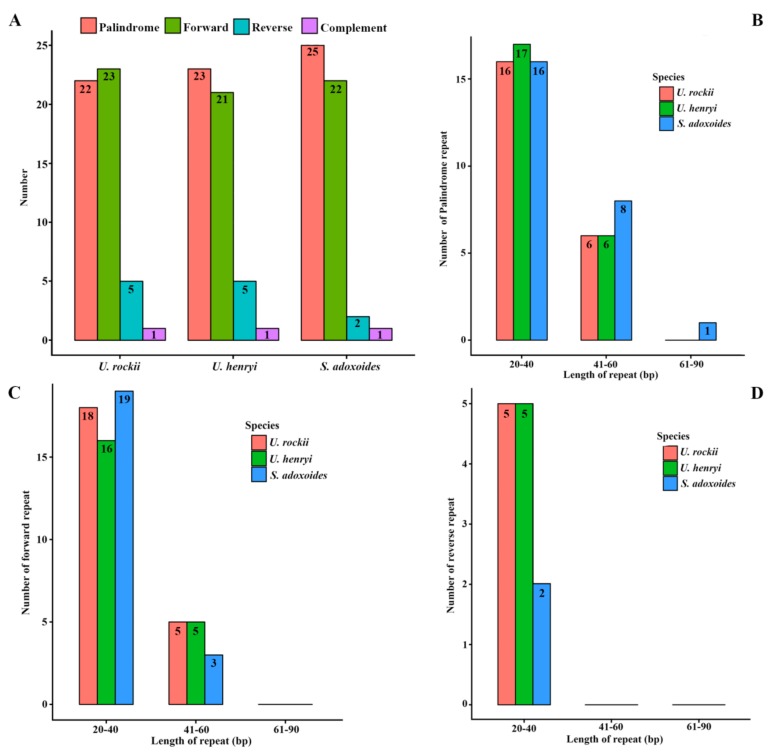
Analysis of repeated sequences in *U. rockii*, *U. henryi*, and *S. adoxoides* chloroplast genomes. (**A**) Total of four repeat types; (**B**) Frequency of the palindromic repeat by length; (**C**) Frequency of the forward repeat by length; (**D**) Frequency of the reverse repeat by length.

Chloroplast repeats are potentially useful genetic resources to investigate population genetics and biogeography of allied taxa [26]. Analyses of various cp genomes revealed that repeat sequences are essential to induce indels and substitutions [27]. Repeat analysis of the *U. rockii* cp genome revealed 22 palindromic repeats, 23 forward repeats, 5 reverse, and 1 complement repeats. Among them, 16 palindromic, 18 forward, and 5 reverse repeats are 20–40 bp in length. Six palindromic and five forward repeats are 41–60 in length (Figure 2). Similarly, 23 and 25 palindromic repeats, 21 and 22 forward repeats, 5 and 2 reverse repeats, and 1 complement repeats were detected, and the detailed repeats length distributions are shown in Figure 2. The number and length of the repeats indicate that *U. rockii* is more similar to *U. henryi* than to *S. aquilegia*. Previous studies suggested that the slipped-strand mispairing and improper recombination of repeat sequences can result in sequence variation and genome rearrangement [28,29,30]. These repeats are informative sources for developing genetic markers for phylogenetic and population studies [31].

**Figure 3:**
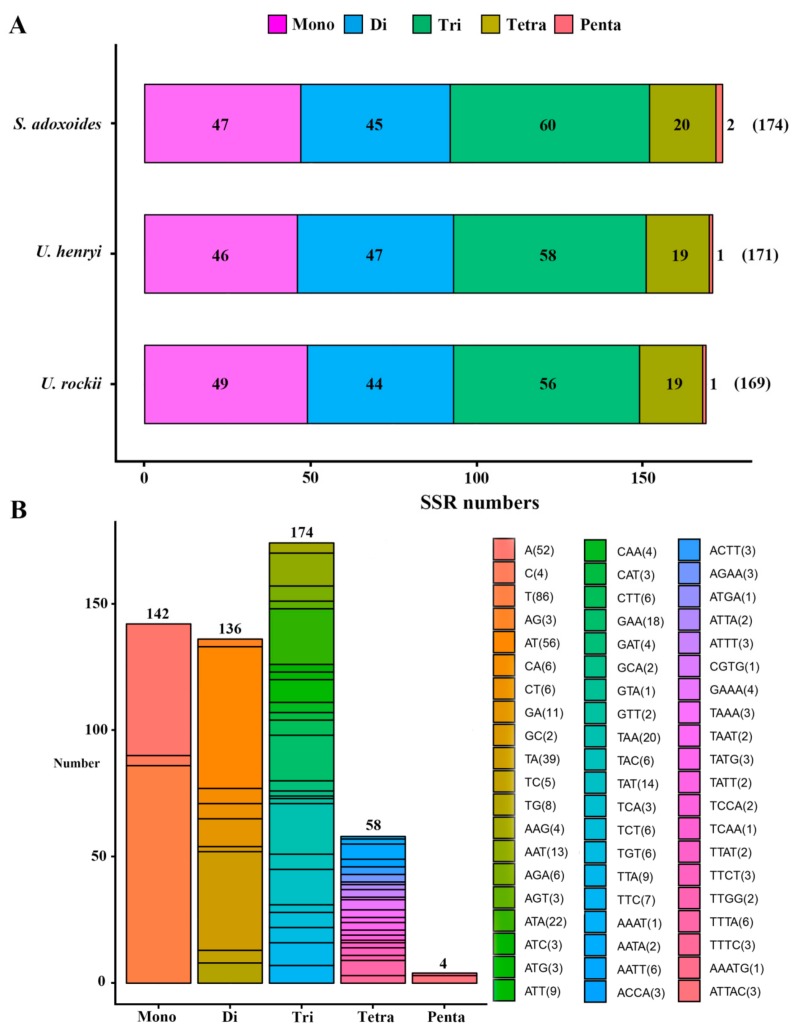
Analysis of simple sequence repeats (SSRs) in chloroplast genomes of the three species. (**A**) Number of different SSR types detected in each species; (**B**) type and frequency of each identified SSR.

Simple sequence repeats (SSRs) in the cp genome can be highly variable at the intra-specific level and are therefore often used as genetic markers in population genetic and evolutionary studies [12,32,33,34]. Because of a high polymorphism rate at the species level, SSRs have been recognized as one of the main sources of molecular markers and have been extensively researched in phylogenetic and biogeographic studies of populations [35,36,37]. In this study, we analyzed the SSRs in the cp genomes. Five categories of perfect SSRs (mono-, di-, tri-, tetra-, and penta-nucleotide repeats) were detected in the cp genome of these three species, with an overall length ranging from 10 to 26 bp (Figure 3, Table S2). Certain parameters were set, because SSRs of 10 bp or longer are prone to slipped-strand mispairing, which is believed to be the main mutational mechanism for polymorphism [38,39,40].

A total of 169 microsatellites were detected in the *U. rockii* cp genome on the basis of the SSR analysis. Similarly, 171 and 174 SSRs were detected in *U. henryi* and *S. adoxoides*, respectively (Figure 3A). The most abundant were tri-nucleotide repeats, which accounted for about 33.85% of the total SSRs, and whose number varies from 56 in *U. rockii* to 60 in *S. adoxoides*, followed by mono-nucleotide repeats (27.63%), di-nucleotide repeats (26.46%), and tetra-nucleotides repeats (11.28%). Penta-nucleotide repeats were the least abundant (0.78%; Figure 3, Table S2). Most previous studies revealed that the richness of SSR types varies between species. In *Quercus* species, mono-nucleotide repeats are the most abundant, accounting for about 80% of the total SSRs [34]. In the cp genome of *Forthysia*, the number of di-nucleotide repeat is the highest [41]. Tri-nucleotide SSRs are most abundant in *Nicotiana species*, accounting for approximately 43.03% [42]. These results suggest that different repeats may contribute to the genetic variations differently among species. Thus, the SSR information will be important for understanding the genetic diversity status of *Urophysa* and its relatives.

In *U. rockii*, more than 96.2% mono-nucleotides are composed of A/T, and a majority of di-nucleotides (84.9%) is composed of A/T (Figure 3B, Table S2), which is consistent with *U. henryi* (97.8% mono-nucleotides and 83.0% di-nucleotides) and *S. aquilegia* (97.9% mono-nucleotides and 85.6% di-nucleotides). Our findings are comparable to previously reported observations that SSRs found in the chloroplast genome are generally composed of poly-thymine (polyT) or poly-adenine (polyA) repeats and infrequently contain tandem cytosine (C) and guanine (G) repeats [43]. Therefore, these SSRs contribute to the AT richness of the three species cp genome, as previously reported for different species [43,44]. SSRs were also detected in CDS regions of the *U. rockii* cp genome. The CDS regions account for approximately 49% of the total length. About 68.6% of SSRs (68.4% for *U. henryi* and 67.2% for *S. adoxoides*) were detected in non-coding regions, whereas only 28.9%of SSRs (29.2% for *U. henryi* and 30.5% for *S. adoxoides*) are present in the protein-coding region of *U. rockii*. Furthermore, about 62.1% of SSRs are present in the LSC region of *U. rockii* (66.1% for *U. henryi* and 68.9% for *S. adoxoides*), and a minority of SSRs exist in IR regions (17.8% in IRa and IRb in total). It was observed that 49 SSRs (28.9%) were located in 19 genes (CDS) regions (*atpF*, *rpoC1*, *rpoC2*, *rps14*, *rps15*, *rps19*, *psaB*, *psaA*, *rbcL*, *rpl33*, *rpl22*, *ndhB*, *ndhD*, *ndhF*, *ndhH*, *ccsA*, *ycf1*, *ycf2*, *ycf3*) in *U. rockii*. The detailed SSR location information is listed in Table S2. These results suggest an uneven distribution of SSRs in the *U. rockii*, *U. henryi*, and *S. adoxoides* cp genomes, as was also reported in different angiosperm cp genomes [44]. Moreover, the cp SSRs of the three species presented abundant variation and are useful for detecting genetic polymorphisms at population, intraspecific, and cultivar levels, as well as for comparing more distant phylogenetic relationships among species.

### 2.3. Genomes Sequence Divergence among the Three Species

**Figure 4:**
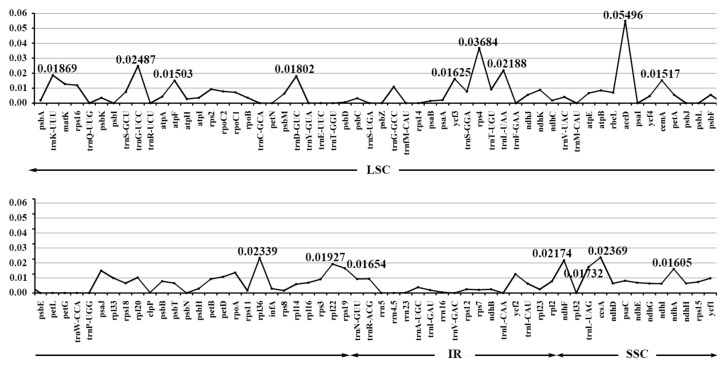
The nucleotide diversity of the whole chloroplast genomes of the three species. LSC: large single-copy region; IRs: inverted repeats region; SSC: small single-copy region.

In order to calculate the sequence divergence level, the nucleotide diversity values in the LSC, SSC, and IR regions of the chloroplast genomes were calculated (Figure 4, Table S3). In the LSC regions, these values varied from 0 to 0.05496, with a mean of 0.00705, in the IR regions they varied from 0 to 0.01265, with a mean of 0.00363, and only the SSC region had >0.010 average sequence nucleotide diversity, and its values varied from 0 to 0.02369, with a mean of 0.01048. All these results indicated that the differences among these genome regions were small. However, some highly variable loci, including *trnK-UUU*, *trnG-UCC*, *trnD-GUC*, *atpF*, *rps4*, *trnL-UAA*, *accD*, *cemA*, *rpl36*, *rpl22*, *rps19*, *ndhF*, *trnL-UAG*, *ccsA*, *ndhA*, and *ycf3* were more precisely located (Figure 4, Table S3). All these regions displayed higher nucleotide diversity values than other regions (value > 0.015). Twelve of these loci were found to be located in the LSC region, and four in the SSC region, but the nucleotide diversity in the IR regions appeared small, less than 0.015. Among these loci, *atpF*, *accD*, *ndhF*, *rpl22*, *ccsA*, and *ycf3* have been detected as highly variable regions in different plants [19,23,45,46]. On the basis of these results, we believe that *accD*, *rps4*, *ccsA*, *rpl36*, and *ndhF*, which have comparatively high sequence deviation, are good sources for interspecies phylogenetic analysis, as shown in previous studies [42,44].

**Figure 5:**
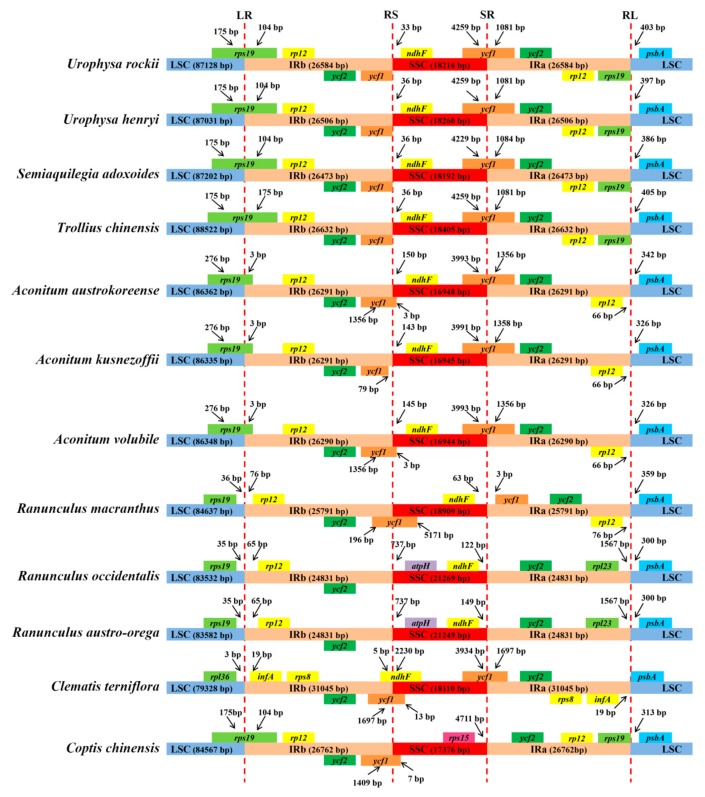
Comparison of the borders of the LSC, SSC, and IR regions of the chloroplast genomes of the three species. LR: junction line between LSC and IRb; RS: junction line between IRb and SSC; SR: junction line between SSC and IRa; RL: junction line between IRa and LSC.

Expansion and contraction at the borders of IR regions is the main reason for size variations in the cp genome and plays a vital role in its evolution [39,47,48]. The IR/LSC and IR/SSC junction regions were compared to identify IR expansion or contraction. The *rps19*, *ndhF*, *ycf1*, and *psbA* genes were located in the junctions of the LSC/IRa, IRa/SSC, SSC/IRb, and IRb/LSC regions, respectively (Figure 5). Despite the similar length of these three species IR regions, from 26,473 to 26,584 bp, some IR expansion and contraction were observed. The *rps19* gene traverses the LSC and IRb regions (LR line), with 104 bp located in the IR region. The RS line (the junction line between IRb and SSC) is located between *ycf1* and *ndhF*, and the variation in distances between the RS line and *ndhF* ranges from 33 to 36 bp across the three species. The SR line (the junction line between SSC and IRa) intersects the ycf1 gene, the SSC and IRa regions are the same in *U. rockii* and *U. henryi* (4259 bp in SSC and 1081 bp in IRb), while different in *S. adoxoides* (4229 bp in SSC and 1084 bp in IRb) (Figure 5). The distance between the *psbA* and RL line varies from 386 to 403 bp. Compared to species of other genera, the IRb/SSC and SSC/IRa regions of *Urophysa* showed an expansion in *ycf1*, but a contraction in *rps19* (Figure 5). The expansion and contraction detected in the IR regions may act as a primary mechanism in creating the length variation of the cp genomes in *U. rockii*, *U. henryi*, and *S. adoxoides*, as previous studies suggested [32,34,42,49].

### 2.4. Phylogenetic Analysis

**Figure 6:**
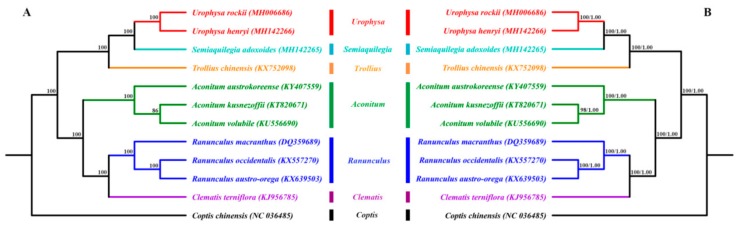
Phylogenetic relationship of *Urophysa* with related species based on 79 single-copy genes shared by all cp genomes. Tree constructed by (**A**) maximum likelihood (ML) with the bootstrap values of ML above the branches; (**B**) maximum parsimony (MP) and Bayesian inference (BI) with bootstrap values of MP and posterior probabilities of BI above the branches, respectively.

To study the phylogenetic position of *U. rockii and U. henryi* within the Ranunculaceae family, we used 79 single-copy genes shared by the cp genomes of 12 Ranunculaceae members, representing seven genera (Figure 6). For Bayesian inference (BI) and maximum parsimony (MP), the posterior probabilities and bootstrap values were very high for each lineage, with all values ≥98%. Both the maximum likelihood (ML), BI, and MP phylogenetic results strongly supported that *U. rockii* is closely clustered with *U. henryi* within the genus *Urophysa*, with *S. adoxoides* as their closest relative with 100% bootstrap value (Figure 6), which is consistent with the results of previous molecular studies [50,51,52]. Furthermore, the species in each genus formed a single clade. The first clade is formed by species of the genera *Urophysa*, *Semiaquilegia*, and *Trollius*, the second clade was divided into two clades: one clade includes the *Ranunculus* and *Clematis* species, and the other clade consists of just the *Aconitum* species. Additionally, the topological structures from the whole complete chloroplast genome sequences and the CDS sequences are similar to that from single-copy genes (Figure S1), and all lineages possess high bootstrap values. These results suggest that there is no conflict among the entire genome data set, CDS sequences, and 79 shared single-copy genes of these cp genomes. Furthermore, these results are in accord with previous phylogeny research [53]. All these phylogenetic analyses are substantially increasing our understanding of the evolutionary relationship among species in Ranunculaceae.

### 2.5. Positive Selected Analysis

**Figure 7:**
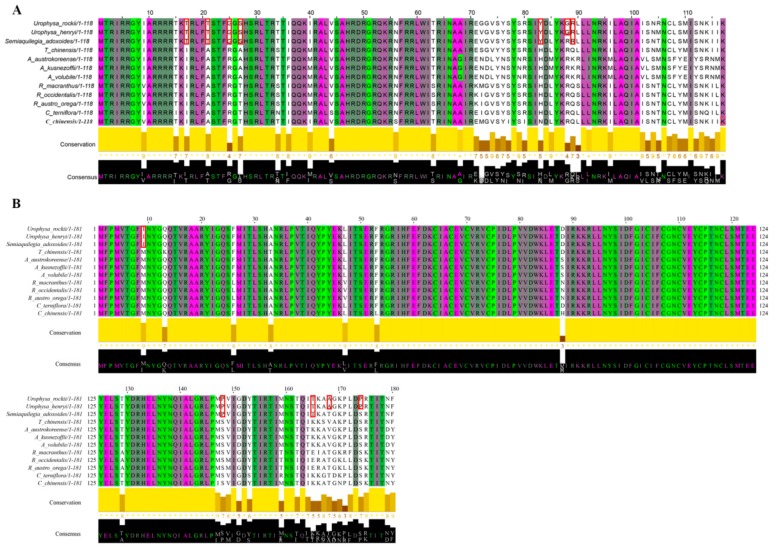
Two of the amino acids sequences that showed positive selection in the branch-site model test. (**A**) Amino acids sequences of the *rpl20* gene; (**B**) amino acids sequences of the *ndhI* gene. The red blocks represent the different amino acids.

Of 57 single-copy CDS genes initially considered for the positive selection analysis (Table S4), 47 were eventually selected (Table 4). No significant positive selection was detected for all genes (*p*-value > 0.05), but six genes that possess high posterior probabilities for codon sites were found in the Bayesian Empirical Bayes (BEB) test (*atpA*, *rpl20*, *psaA*, *atpB*, *ndhI*, and *rbcL*) (Figure 7, Figure S2 and Table 4). Previous studies suggested that codon sites with a high posterior probability should be regarded as positively selected sites [54], which means that these six genes may be under positive selection pressure [55]. After Jalview visualization, the results of the amino acid properties across each column of all species revealed that many amino acids vary between different genera, such as the 88th amino acid (G in *U. rockii* and *U. henryi*, R in other species) of the *rpl20* gene (Figure 7A) and other amino acids (marked with red blocks in Figure 7A). In the *ndhI* gene, two amino acids (the A in 168th and the P in 174th) were specific for *U. rockii* and *U. henryi*, and three amino acids (the 9th, 148th, and 165th, marked with red blocks in Figure 7B) were only possessed by *U. rockii*, *U. henryi*, and *S. adoxoides*. The amino acid properties of the other four genes (*atpA*, *atpB*, *rbcL*, and *psaA*) are shown in Figure S2. As we know, most amino acids may be under strong structural and functional constraints and not free to change [55]. We detected six genes with high posterior probability in codon site and many different amino acids among species, which may play an important role in *Urophysa* species evolution and environment adaptation. Populations of *U. rockii* and *U. henryi* are distributed only in karst regions of southern China, and the karst environments are characterized by low soil water content, insufficient light, and poor nutrient availability, which might have exerted strong selective forces on plant evolution [56].

However, five of the abovementioned six genes are involved in photosynthesis (*atpA*, *psaA*, *atpB*, *ndhI*, and *rbcL*) (Table 3). The gene *rpl20* is involved in translation, which is an important part of protein synthesis [57]. The genes *atpA* and *atpB* participate in ATP synthesis, which is the main source of energy for the functioning of living cells and all multicellular organisms [58]. Additionally, *rbcL* is the gene for the Rubisco large subunit protein, which is an important component of photosynthetic electron transport [59,60]. Most previous research has revealed that positive selection of the *rbcL* gene in land plants may be a common phenomenon [61]. All these genes might play important roles when founder effects occur in populations; both changes in selection pressures and genetic drift result in the rapid shift of these genes to a new, coadapted combination. Therefore, all these genes under positive selection give an indication of why *U. rockii* and *U. henryi* could adapt to the harsh environment of karst (characterized by low soil water content, periodic water deficiency, and poor nutrient availability). Moreover, the results of the gene effectiveness test (*rbcL* and *rpl20*) (Figure S3) suggested that these genes can distinguish the species of *Urophysa* and its relatives and can be used for future phylogenetic analyses. The six genes will not only provide insights into chloroplast genome evolution of species of *Urophysa*, but also offer valuable genetic markers for population phylogenomic studies of *Urophysa* and its close lineages.

## 3. Materials and Methods

### 3.1. Plant Materials and DNA Extraction

Fresh leaves of *U. rockii*, *U. henryi*, and *S. aquilegia* were collected from Jiangyou (Sichuan, China; coordinates: 31°59′ N, 104°51′ E), Yichang (Hubei, China; coordinates: 30°42′ N, 111°17′ E), and Nanchuan (Chongqing, China; coordinates: 30°04′ N, 90°33′ E), respectively. The fresh leaves from each site were immediately dried with silica gel for further DNA extraction. The total genomic DNA was extracted from leaf tissues with a modified Cetyl Trimethyl Ammonium (CTAB) method [62].

### 3.2. Chloroplast Genome Sequencing and Assembling

All cp genomes were sequenced using an Illumina Hiseq 2500 platform by Biomarker Technologies, Inc. (Beijing, China) In order to eliminate the interference from mitochondrial or nuclear DNAs, all the cp genome reads were extracted by mapping all raw reads to the reference cp genome of *Trollius chinensis* (KX752098) with Burrows Wheeler Alignment (BWA) [63]. High-quality reads were obtained using the CLC Genomics Workbench v7.5 (CLC Bio, Aarhus, Denmark) with the default parameters set. A few gaps in the assembled cp genomes were corrected by Sanger sequencing. The primers were designed using Lasergene 7.1 (DNASTAR, Madison, WI, USA). Primer synthesis and the sequencing of the polymerase chain reaction products were conducted by Sangon Biotech (Shanghai, China). The primers and amplifications are shown in Supplementary Table S5.

### 3.3. Genome Annotation and Analysis

The complete cp genomes were annotated using the online program DOGMA [64]. The annotation results were checked manually, and the codon positions were adjusted by comparing to a previously homologous gene from various chloroplast genomes present in the database using Geneious R11 (Biomatters, Ltd., Auckland, New Zealand). Furthermore, the OGDRAW1 program [65] was used to draw the circular plastid genome maps. GC content and codon usage were analyzed by the MEGA 6 software [66]. The complete cp genomes of *U. rockii*, *U. henryi*, and *S. adoxoides* are deposited in the GenBank under the accession numbers MH006686, MH142266, and MH142265, respectively.

### 3.4. Repeat Sequence Characterization and SSRs

Perl script MISA [67] was used to search for microsatellites (mono-, di-, tri-, tetra-, penta-, and hexa-nucleotides) loci in the cp genomes. The minimum numbers (thresholds) of the SSRs were 10, 5, 4, 3, 3, and 3 for mono-, di-, tri-, tetra-, penta-, and hexa-nucleotides, respectively. All the repeats were manually verified, and redundant results were removed. REPuter was employed to identify repeat sequences, including palindromic, forward, reverse, and complement, within the cp genome [68]. The following conditions for repeat identification were used: (1) Hamming distance of 3; (2) 90% or greater sequence identity; (3) a minimum repeat size of 30 bp.

### 3.5. Phylogenetic Analysis

Phylogenetic analysis was conducted using the single-copy genes of the three taxa, together with nine species downloaded from the NCBI GenBank (Tables S6 and S7). The sequences were aligned using MAFFT v5 [69] in GENEIOUS R11 (Biomatters, Ltd.) with the default parameters set and were manually adjusted in MEGA 6.0 [66]. Maximum parsimony (MP) analyses were conducted using PAUP [70]. All characters were equally weighted, gaps were treated as missing, and character states were treated as unordered. Heuristic search was performed with MULPARS option, tree bisection-reconnection (TBR) branch swapping, and random stepwise addition with 1000 replications. The maximum likelihood (ML) analyses were performed using RAxML 8.0 [71]. For ML analyses, the best-fit model, general time reversible (GTR) + G was used with 1000 bootstrap replicates. Bayesian inference (BI) was performed with Mrbayes v3.2 [72]. The Markov chain Monte Carlo (MCMC) analysis was run for 1 × 10^8^ generations. The trees were sampled at every 1000 generations with the first 20% discarded as burn-in. The remaining trees were used to build a 50% majority-rule consensus tree. The stationarity was considered to be reached when the average standard deviation of split frequencies remained below 0.001. Additionally, in order to test the utility of different cp regions, phylogenetic analyses were performed for the complete chloroplast genome sequences and the CDS sequences, respectively.

### 3.6. Chloroplast Genome Nucleotide Diversity and Positive Selected Analysis

The cp genome sequences were aligned using MAFFT v5 [69] and adjusted manually. Furthermore, a sliding window analysis was conducted for nucleotide diversity in LSC, SSC, and IR regions of the cp genomes using the DnaSP version 5.1 [73]. In addition, to identify the genes under positive selection in *U. rockii* and *U. henryi*, endemic to special karst environment, an optimized branch-site model [74] combined with Bayesian Empirical Bayes (BEB) methods [55] were used by comparison with their relatives. We firstly extracted all CDS sequences from *U. rockii*, *U. henryi*, *S. adoxoides*, and nine closely related species downloaded from GenBank (Table S6). The single-copy CDS sequences between these twelve species were obtained (see the Table S4). Each single-copy CDS sequence of these twelve species was aligned according to their amino acid sequence alignment generated by MUSCLE [75], and the “number of gaps” in the alignments was further checked. Then, the alignments of the corresponding DNA codon sequences were further trimmed by TRIMAL [76], and the bona fide alignments were used to support the subsequent positive selection analysis. The optimized branch-site model in the CODEML program implemented in the PAML 4 package [77] was used to assess potential positive selection affecting individual codons along a specifically designated lineage, which was set as *U. rockii* and *U. henryi*. Selective pressure is measured by the ratio (*ω*) of the nonsynonymous substitution rate (dN) to the synonymous substitutions rate (dS). A ratio *ω* > 1 indicates positive selection, *ω* = 1 implies neutral selection, and *ω* < 1 suggests negative selection [78]. Log-likelihood values were calculated in an alternative branch-site model (Model = 2; NSsites = 2; and Fix = 0) that allowed *ω* to vary among different codons along particular lineages and a neutral branch-site model (Model = 2; NSsites = 2; Fix = 1; Fix *ω* = 1) that confined the codon sites under neutral selection (*ω* = 1) on the basis of the likelihood ratio tests (LRT). The right-tailed chi-square test was performed to calculate the p values based on the difference in log-likelihood values between the alternative model and the neutral model with one degree of freedom to assess the model fit. Then, the p values were further adjusted according to multiple statistical tests [79]. A gene with an adjusted p value smaller than 0.05 and with positively selected sites was considered a positively selected gene (PSG). Moreover, in order to identify specific amino acid sites that are potentially under positive selection, a BEB method was implemented to calculate the posterior probabilities for sites classes. Codon sites with a high posterior probability were regarded as positively selected sites [54]. Jalview [80] was used to view the amino acid sequences of positively selected genes. In the end, in order to test the effectiveness of genes under positive selection, we randomly chose two genes to conduct the phylogenetic analyses.

## Figures and Tables

**Table 1:** Summary of complete chloroplast genomes. LSC, large single-copy; SSC, small single-copy; IR, inverted repeat

Species	LSC			SSC			IR			Total	
	Length (bp)	GC%	Length (%)	Length (bp)	GC%	Length (%)	Length (bp)	GC%	Length (%)	Length (bp)	GC%
*U. rockii*	87,128	37.2	55.0	18,216	32.5	11.5	26,584	43.7	16.8	158,512	38.8
*U. henryi*	87,031	37.2	55.0	18,260	32.6	11.5	26,506	43.6	16.7	158,303	38.8
*S. adoxoides*	87,202	37.2	55.1	18,192	32.5	11.5	26,473	43.7	16.7	158,340	38.9
*Tsuga chinensis*	88,522	36.3	55.3	18,405	32.0	11.5	26,632	43.1	16.6	160,191	38.1
*Aconitum austrokoreense*	86,362	36.2	55.4	16,948	32.7	10.9	26,291	43.0	16.9	155,892	38.1
*A. kusnezoffii*	86,335	36.2	55.4	16,945	32.7	10.9	26,291	43.0	16.9	155,862	38.1
*A. volubile*	86,348	36.2	55.4	16,944	32.6	10.9	26,290	43.0	16.9	155,872	38.1
*Ranunculus macranthus*	84,637	36.0	54.6	18,909	31.0	12.2	25,791	43.5	16.6	155,129	37.9
*R. occidentalis*	83,532	35.9	54.1	21,269	31.6	13.8	24,831	43.6	16.1	154,474	37.8
*R. austro-oreganus*	83,582	35.9	54.1	21,249	31.6	13.8	24,831	43.6	16.1	154,493	37.8
*Clematis terniflora*	79,328	36.3	49.7	18,110	31.4	11.4	31,045	42.0	19.5	159,528	38.0
*Coptis chinensis*	84,567	36.4	54.4	17,376	32.1	11.2	26,762	43.0	17.2	155,484	38.2

**Table 2:** Comparison of the sizes of coding and non-coding regions among species.

Species	Protein-Coding			tRNA			rRNA		
	Length (bp)	GC%	Length (%)	Length (bp)	GC%	Length (%)	Length (bp)	GC%	Length (%)
*U. rockii*	78,867	39.2	49.8	2687	53.2	1.7	8602	55.8	5.4
*U. henryi*	78,769	39.2	49.8	2695	53.3	1.7	8602	55.8	5.4
*S. adoxoides*	78,498	39.3	49.6	2706	53.6	1.7	8602	55.8	5.4
*T. chinensis*	78,903	38.4	49.3	2716	53.1	1.7	9050	55.4	5.6
*A. austrokoreense*	79,575	38.3	51.0	2810	53.0	1.8	9050	55.4	5.8
*A. kusnezoffii*	78,294	38.4	50.2	2813	52.9	1.8	9046	55.3	5.8
*A. volubile*	79,560	38.3	51.0	2810	53.0	1.8	9050	55.5	5.8
*R. macranthus*	78,615	38.2	50.7	2738	53.1	1.8	7559	55.2	4.9
*R. occidentalis*	69,294	38.6	44.9	2717	53.1	1.8	9050	55.4	5.9
*R. austro-oreganus*	74,355	38.1	48.1	2796	52.9	1.8	9050	55.4	5.9
*C. terniflora*	81,819	38.3	51.3	2718	53.4	1.7	9050	55.4	5.7
*C. chinensis*	71,637	39.0	46.1	2716	53.2	1.7	9050	55.5	5.8

**Table 3:** List of genes encoded in two *Urophysa* species and *S. adoxoides.*

Category for Genes	Group of Genes	Name of Genes
Self-replication	transfer RNAs	trnA-UGC *, *trnC-GCA*, *trnD-GUC*, *trnE-UUC*, *trnF-GAA*, *trnfM-CAU*, *trnG-GCC*, *trnG-UCC*, trnI-CAU *, trnI-GAU *, *trnK-UUU*, trnL-CAA *, *trnL-UAA*, *trnL-UAG*, *trnM-CAU*, trnN-GUU *, *trnP-UGG*, *trnQ-UUG*, trnR-ACG *, *trnR-UCU*, *trnS-GCU*, *trnS-GGA*, *trnS-UGA*, *trnT-GGU*, *trnT-UGU*, trnV-GAC *, *trnV-UAC*, *trnW-CCA*, *trnY-GUA*
	ribosomal RNAs	rrn4.5 *, rrna5 *, rrn16 *, rrn23 *
	RNA polymerase	*rpoA*, *rpoB*, *rpoC1*, *rpoC2*
	Small subunit of ribosomal proteins (SSU)	*rps2*, *rps3*, *rps4*, rps7 *, *rps8*, *rps11*, rps12 *, *rps14*, *rps15*, *rps16*, *rps18*, rps19 *
	Large subunit of ribosomal proteins (LSU)	rpl2 *, *rpl14*, *rpl16*, *rpl20*, *rpl22*, rpl23 *, *rpl32*, *rpl33*, *rpl36*
Genes for photosynthesis	Subunits of NADH-dehydrogenase	*ndhA*, ndhB *, *ndhC*, *ndhD*, *ndhE*, *ndhF*, *ndhG*, *ndhH*, *ndhI*, *ndhJ*, *ndhK*
	Subunits of photosystem I	*psaA*, *psaB*, *psaC*, *psaI*, *psaJ*
	Subunits of photosystem II	*psbA*, *psbB*, *psbC*, *psbD*, *psbE*, *psbF*, *psbH*, *psbI*, *psbJ*, *psbK*, *psbL*, *psbM*, *psbN*, *psbT*, *psbZ*
	Subunits of cytochrome b/f complex	*petA*, *petB*, *petD*, *petG*, *petL*, *petN*
	Subunits of ATP synthase	*atpA*, *atpB*, *atpE*, *atpF*, *atpH*, *atpI*
	Large subunit of rubisco	*rbcL*
Other genes	Tanslational initiation factor	*infA*
	Protease	*clpP*
	Maturase	*matK*
	Subunit of Acetyl-CoA-carboxylase	*accD*
	Envelope membrane protein	*cemA*
	C-type cytochrome synthesis gene	*ccsA*
Genes of unknown function	hypothetical chloroplast reading frames (ycf)	ycf1 *, ycf2 *, *ycf3*, *ycf4*

* Gene with two copies.

**Table 4:** The potential positive selection test based on the branch-site model.

Gene Name	Null Hypothesis			Alternative Hypothesis			Significance Test		
	lnL	df	Omega (*ω* = 1)	lnL	df	Omega (*ω* > 1)	BEB	NEB	*p*-Value
*psbI*	−188.6475	26	1	−188.6475	27	3.40383	NA	NA	1
*psbL*	−164.11693	26	1	−164.1169	27	3.40719	NA	NA	1
*rps14*	−621.64162	26	1	−621.6416	27	3.40833	NA	NA	1
*psaI*	−214.67663	26	1	−214.6766	27	3.38764	NA	NA	1
*atpH*	−434.45059	26	1	−434.4506	27	3.35869	NA	NA	1
*psaJ*	−318.52192	26	1	−318.5219	27	3.4089	NA	NA	1
*atpE*	−868.20243	26	1	−868.2024	27	3.40891	NA	NA	1
*atpA*	−3297.629	26	1	−3297.41	27	69.43581	220, E, 0.794	NA	5.04 × 10^-1^
*petN*	−126.25816	26	1	−126.2582	27	3.40693	NA	NA	1
*rps11*	−920.92455	26	1	−920.9246	27	1	NA	NA	1
*psbT*	−216.52331	26	1	−216.5233	27	1	NA	NA	1
*ndhG*	−1238.1161	26	1	−1238.116	27	3.33667	NA	NA	9.99 × 10^-1^
*ycf4*	−1275.4093	26	1	−1275.409	27	3.40886	NA	NA	1
*rps18*	−567.98294	26	1	−567.9829	27	3.39414	NA	NA	1
*petB*	−1274.0507	26	1	−1274.051	27	3.403	NA	NA	1
*rpl20*	−1000.285	26	1	−999.941	27	112.30316	88, R, 0.683	NA	4.07 × 10^-1^
*psbN*	−223.7602	26	1	−223.7602	27	3.40292	NA	NA	1
*psbF*	−198.46733	26	1	−198.4673	27	3.38407	NA	NA	1
*petG*	−206.74878	26	1	−206.7488	27	3.42095	NA	NA	1
*psbK*	−375.13705	26	1	−375.1371	27	3.4063	NA	NA	1
*rpl36*	−267.8099	26	1	−267.8099	27	1	NA	NA	1
*rps2*	−1620.734	26	1	−1620.734	27	3.40891	NA	NA	1
*psbM*	−179.71897	26	1	−179.719	27	3.4064	NA	NA	1
*rpoB*	−6830.0894	26	1	−6830.089	27	3.40847	NA	NA	9.99 × 10^-1^
*psaA*	−4245.754	26	1	−4245.49	27	63.47379	28, R, 0.778	NA	4.66 × 10^-1^
*psbH*	−540.92362	26	1	−540.9236	27	3.40123	NA	NA	1
*ndhE*	−616.75534	26	1	−616.7553	27	3.40218	NA	NA	1
*atpB*	−3133.747	26	1	−3133.75	27	1	115, N, 0.828	NA	1
*ndhI*	−1307.986	26	1	−1307.68	27	575.22179	174, S, 0.696	NA	4.35 × 10^-1^
*cemA*	−1787.561	26	1	−1787.561	27	3.40891	NA	NA	1
*ndhJ*	−1001.4075	26	1	−1001.407	27	1	NA	NA	1
*psbJ*	−209.10513	26	1	−209.1051	27	3.38566	NA	NA	1
*petA*	−1331.3789	26	1	−1331.379	27	3.4089	NA	NA	1
*psbC*	−2760.6743	26	1	−2760.674	27	1	NA	NA	1
*ndhH*	−2643.2896	26	1	−2643.29	27	1	NA	NA	9.98 × 10^-1^
*rbcL*	−2937.477	26	1	−2937.41	27	5.22178	440, E, 0.736	NA	7.20 × 10^-1^
*clpP*	−1301.1173	26	1	−1301.117	27	3.40876	NA	NA	1
*ndhC*	−731.03212	26	1	−731.0321	27	3.33544	NA	NA	1
*ycf3*	−935.76375	26	1	−935.7638	27	3.40891	NA	NA	1
*psbD*	−1922.7755	26	1	−1922.775	27	3.38592	NA	NA	1
*psbA*	−1960.3785	26	1	−1960.379	27	3.39639	NA	NA	1
*petL*	−172.24809	26	1	−172.2481	27	3.40087	NA	NA	1
*rpl33*	−413.59385	26	1	−413.5939	27	3.4089	NA	NA	1
*psbE*	−435.90511	26	1	−435.9051	27	3.40785	NA	NA	1
*psaC*	−498.98549	26	1	−498.9855	27	3.408	NA	NA	1
*atpI*	−1445.5558	26	1	−1445.556	27	3.39588	NA	NA	1
*psaB*	−4069.2947	26	1	−4069.295	27	3.41513	NA	NA	1

Bold types are positively selected sites. BEB: Bayesian Empirical Bayes; NEB: Naïve Empirical Bayes; Amino acid: (E: Glu; R: Arg; N: Asn; S: Ser).

## Supplementary Materials

Supplementary Materials are available online at http://www.mdpi.com/1422-0067/19/7/1847/s1.
