# Supplementary material for: Comparative Analysis of the Chloroplast Genomes of the Chinese Endemic Genus Urophysa and Their Contribution to Chloroplast Phylogeny and Adaptive Evolution
Source: Int J Mol Sci. 2018 Jun 22;19(7):1847. doi: 10.3390/ijms19071847 (PMC6073864; doi:10.3390/ijms19071847)
Supplement: Supplementary file 1 [file ijms-19-01847-s001.zip › Supplementary Materials/Table S1 The genes with introns and exons in the U. rockii chloroplast genome and their locations.docx]

**Table S1 The genes with introns and exons in the *U. rockii* chloroplast genome and their locations.**

| **Gene** | **Location** | **Exon I (bp)** | **Intron I (bp)** | **Exon II (bp)** | **Intron II (bp)** | **Exon III (bp)** |
| --- | --- | --- | --- | --- | --- | --- |
| *rps16* | LSC | 227 | 866 | 40 |  |  |
| *atpF* | LSC | 410 | 693 | 145 |  |  |
| *rpoC1* | LSC | 1613 | 723 | 430 |  |  |
| *ycf3* | LSC | 159 | 755 | 230 | 712 | 124 |
| *clpP* | LSC | 246 | 653 | 289 | 809 |  |
| *rpl2* | IRa | 434 | 654 | 391 |  |  |
| *rpl2* | IRb | 434 | 654 | 391 |  |  |
| *ndhB* | IRa | 756 | 715 | 777 |  |  |
| *ndhB* | IRb | 756 | 715 | 777 |  |  |
| *ndhA* | SSC | 536 | 849 | 553 |  |  |
