# Supplementary material for: Comparative Analysis of the Chloroplast Genomes of the Chinese Endemic Genus Urophysa and Their Contribution to Chloroplast Phylogeny and Adaptive Evolution
Source: Int J Mol Sci. 2018 Jun 22;19(7):1847. doi: 10.3390/ijms19071847 (PMC6073864; doi:10.3390/ijms19071847)
Supplement: Supplementary file 1 [file ijms-19-01847-s001.zip › Supplementary Materials/Table S5 Primers used for gap closure in this study.docx]

**Table S5. Primers used for gap closure in this study.**

| **Primer** | **Direction** | **Sequence** | **Production size (bp)** | **Tm** |
| --- | --- | --- | --- | --- |
| 1 | F | GGCGCATACCTAAACGGAAACTAA | 345 | 60 |
|  | R | GAACCATCCAATGTAAAGACGGTT |  |  |
| 2 | F | CTGCCTTCTTTGAAATATCA | 733 | 60 |
|  | R | TCCCGTGTGATACACTTTTG |  |  |
| 3 | F | CGAATCAAGATACCCATT | 118 | 60 |
|  | R | GACCTCTTGCCAATTGAT |  |  |
| 4 | F | CCGCGTAATATATGCACT | 572 | 58 |
|  | R | GGGCGCTATACTGGATCT |  |  |
| 5 | F | TGGCTCGTTCCGCCATCCC | 593 | 60 |
|  | R | TAACCAAACCAACGAGTCA |  |  |
| 6 | F | TGAGTAACGCAAACATTGGT | 938 | 58 |
|  | R | CTCGAATTCTAACCTTGTGT |  |  |
| 7 | F | TGTGATAGGAATGAACAGGAAC | 268 | 56 |
|  | R | AATTCTTCCTCGGCTTCGGGTA |  |  |
| 8 | F | TGACATCTCTTGATCTGCA | 164 | 56 |
|  | R | GTGTCGGGTACTGCATGGT |  |  |

The PCR program began with 4-min initial denaturing at 94 ºС followed by 30 cycles of 1-min denaturation at 94 ºС, 1-min annealing at abovementioned Tm, and 2-min extension at 72 ºС, a final extension was run for 7 min at 72 ºС.
