# Supplementary material for: Comparative Analysis of the Chloroplast Genomes of the Chinese Endemic Genus Urophysa and Their Contribution to Chloroplast Phylogeny and Adaptive Evolution
Source: Int J Mol Sci. 2018 Jun 22;19(7):1847. doi: 10.3390/ijms19071847 (PMC6073864; doi:10.3390/ijms19071847)
Supplement: Supplementary file 1 [file ijms-19-01847-s001.zip › Supplementary Materials/Table S6 cp genome sequences of outgroups from Genbank used this study.docx]

**Table S6 cp genome sequences of outgroups from GenBank used in this study.**

| **Species** | **GenBank accession numbers** |
| --- | --- |
| *Trollius chinensis* | KX752098 |
| *Aconitum austrokoreense* | KY407559 |
| *Aconitum kusnezoffii* | KT820671 |
| *Aconitum volubile* | KU556690 |
| *Ranunculus macranthus* | DQ359689 |
| *Ranunculus austro orega* | KX557270 |
| *Ranunculus occidentalis* | KX639503 |
| *Clematis terniflora* | KJ956785 |
| *Coptis chinensis* | NC_036485 |
