# Supplementary figures and images for: Comparative Analysis of the Chloroplast Genomes of the Chinese Endemic Genus Urophysa and Their Contribution to Chloroplast Phylogeny and Adaptive Evolution
Source: Int J Mol Sci. 2018 Jun 22;19(7):1847. doi: 10.3390/ijms19071847 (PMC6073864; doi:10.3390/ijms19071847)

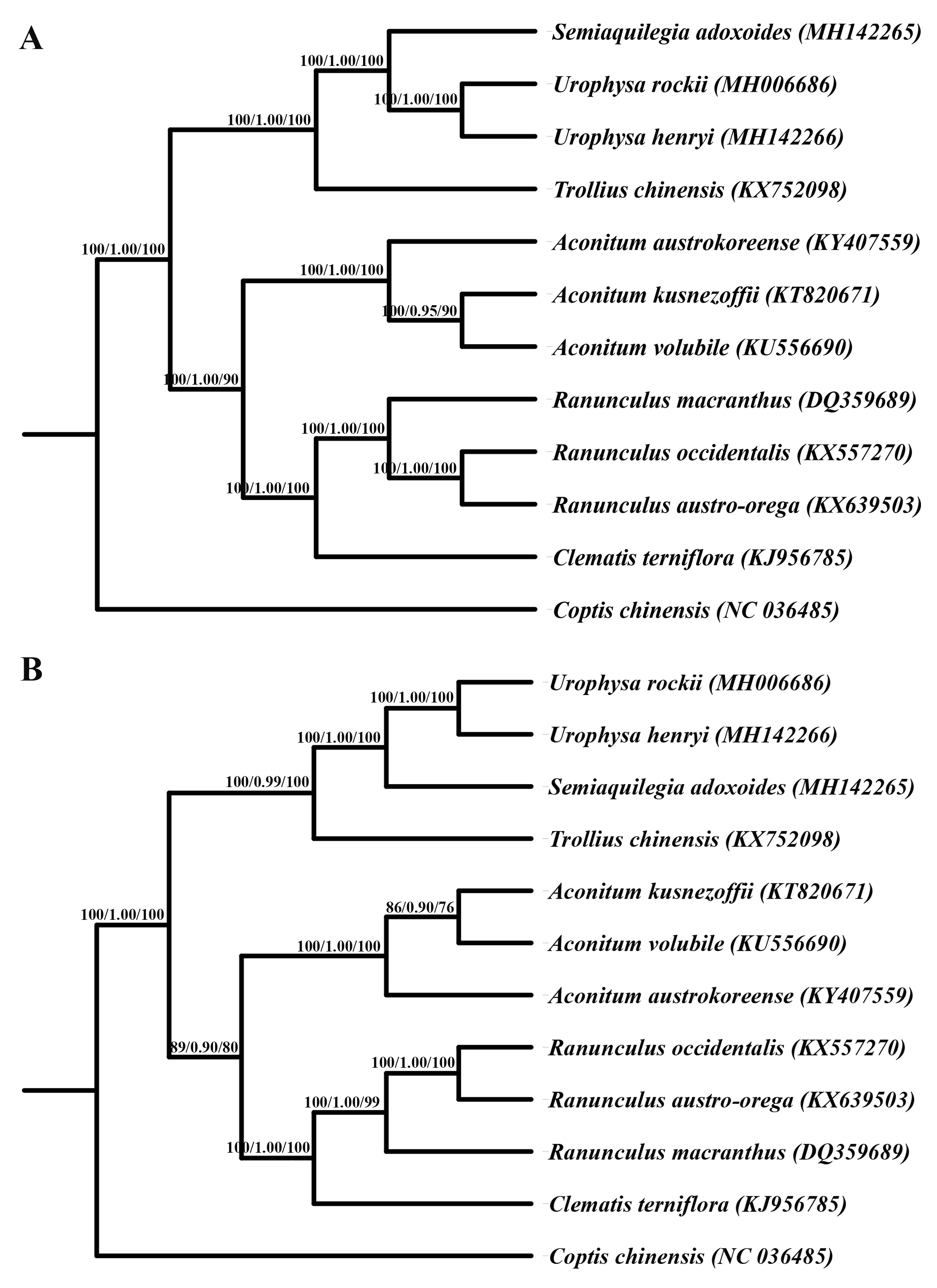

Supplement: Supplementary file 1 [file ijms-19-01847-s001.zip › Supplementary Materials/Figure S1 Phylogenetic tree reconstruction of 12 taxa based on (A) the whole cp genome sequences and (B) the CDS sequences.tif]

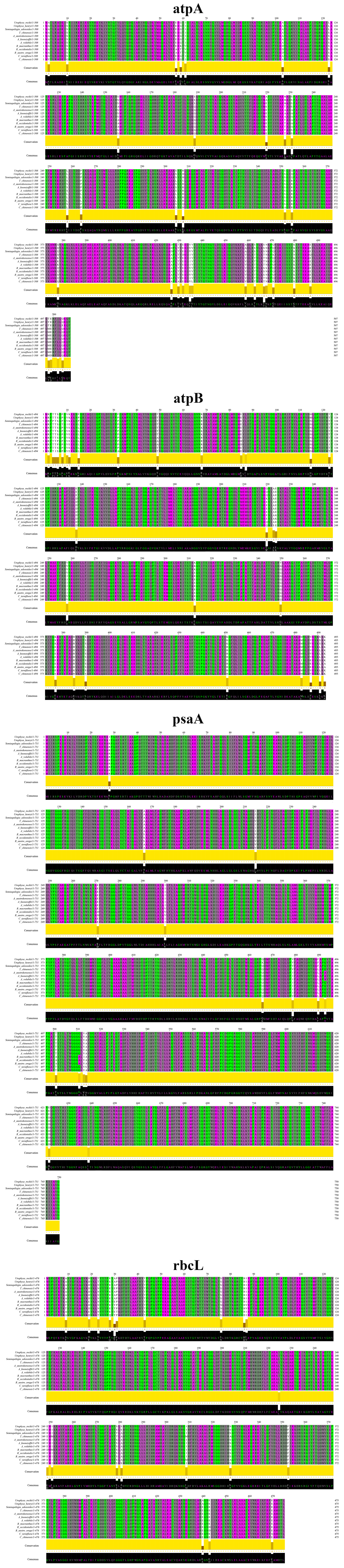

Supplement: Supplementary file 1 [file ijms-19-01847-s001.zip › Supplementary Materials/Figure S2 Four amino acids sequences (atpA, atpB, psaA and rbcL) that shown positive selection in branch-site model test..tif]

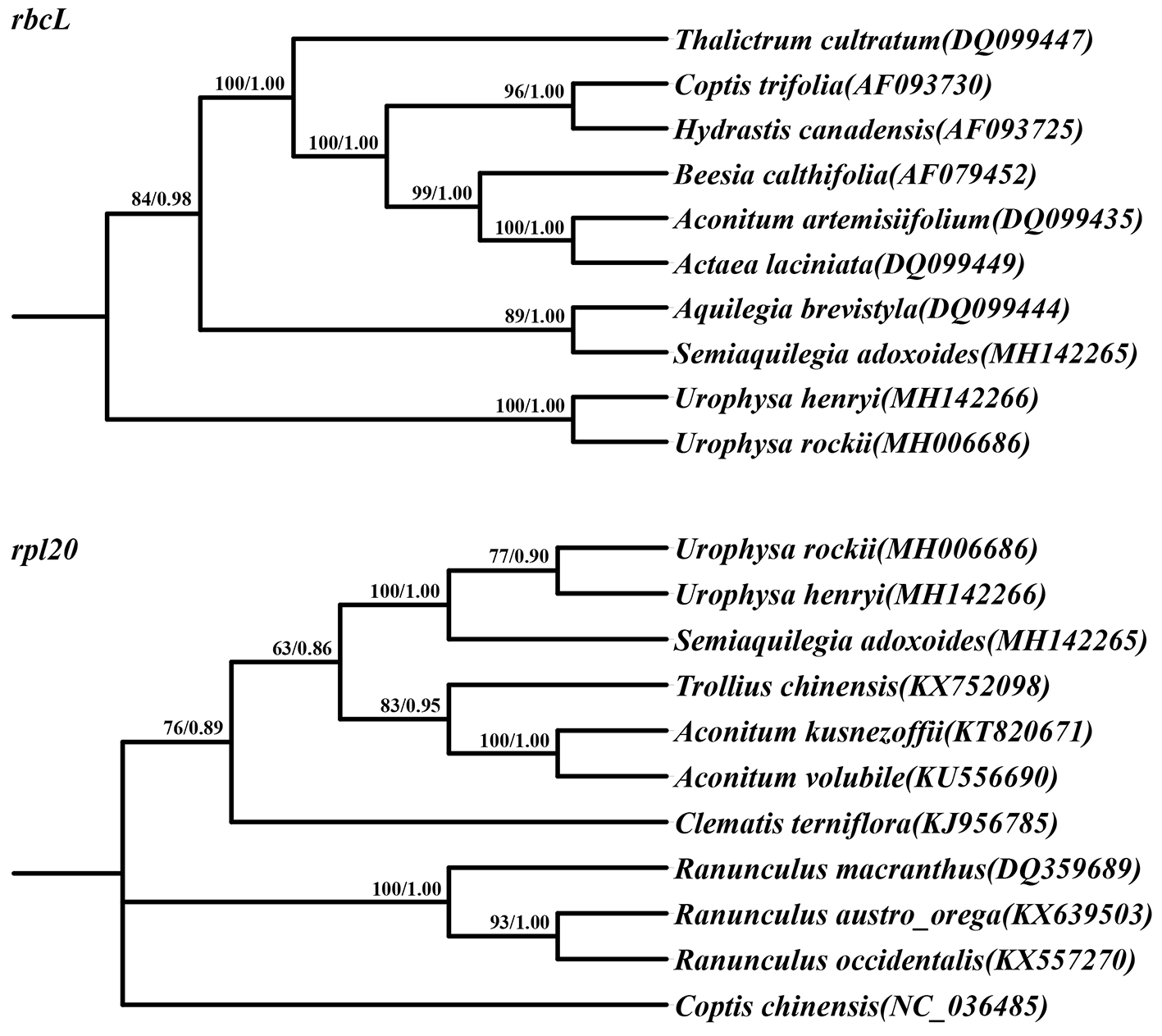

Supplement: Supplementary file 1 [file ijms-19-01847-s001.zip › Supplementary Materials/Figure S3 The effectiveness test of rbcL and rpl20 in the phylogenetic analyses.tif]
